# Measurement of Cognitive and Kinematic Adaptation in Exoskeleton-Assisted Locomotion: Validation of an XR-Based Framework

**DOI:** 10.3390/s26123635

**Published:** 2026-06-07

**Authors:** Nicola Abeni, Riccardo Costa, Emilia Scalona, Diego Torricelli, Matteo Lancini

**Affiliations:** 1 Department of Mechanical and Industrial Engineering, University of Brescia, 25123 Brescia, Italy; r.costa007@studenti.unibs.it; 2 Istituto Nazionale per l’Assicurazione contro gli Infortuni sul Lavoro (INAIL) Prosthetic Centre, Vigorso di Budrio, 40054 Bologna, Italy; e.scalona@inail.it; 3 BioRobotics Group, Centre for Automation and Robotics (CAR), Consejo Superior de Investigaciones Científicas (CSIC), 28500 Madrid, Spain; diego.torricelli@csis.es; 4 Department of Medical and Surgical Specialties, Radiological Sciences, and Public Health, University of Brescia, 25123 Brescia, Italy; matteo.lancini@unibs.it

**Keywords:** exoskeleton, kinematics, cognitive load, visuospatial attention, rehabilitation, mixed reality, wearable sensors, eye-tracking, assisted locomotion

## Abstract

Robotic assistive devices, such as exoskeletons, are increasingly employed in walking rehabilitation. Therefore, the measurement of both movement kinematics and cognitive workload is important to understand this human–robot interaction in real-world contexts. To address this need this study presents the validation of a framework integrating inertial motion capture (Xsens) and eye-tracking sensor (Pupil Neon) within a Mixed Reality (Meta Quest 3) architecture. We developed an overground dual-task paradigm in which holographic numbers appear in the user’s peripheral vision. This setup actively stimulates visuospatial attention while quantifying kinematic and cognitive output. To validate the framework, the protocol has been tested on 30 healthy subjects across repeated exoskeleton training sessions. Statistical analyses revealed that the Coefficient of Multiple Correlation (CMC) and Spectral Arc Length (SPARC), calculated on the shank angular velocity, together with the Step Length Variability, exhibited significant time effects (*p* < 0.01), mapping the transition toward automated gait. Concurrently, pupillometric data demonstrated a measurable reduction in neurocognitive demand; specifically, the Task-Evoked Pupillary Response (TEPR) decreased significantly across progressive training sessions (*p* < 0.05). With this work, we validated a measurement protocol that aims to provide a novel methodology for objectively evaluating motor and cognitive adaptation in wearable assistive devices.

## 1. Introduction

The ability to move freely is essential for performing everyday tasks. This significantly influences human life quality, well-being, and social engagement. However, injuries such as spinal cord injuries (SCIs), neurological disorders, stroke, and cerebral palsy can severely limit walking mobility. Consequently, the demand for assistive technology is increasing [1], leading to a growing interest in advanced devices. Over the past decade, the research on robotic exoskeletons has experienced significant growth. Hardware and energy sources have been improved and have led to the development of functional prototypes suitable for human testing [2,3]. Nowadays, lower-limb exoskeletons are generally divided into three categories: the ones which are designed to enhance the physical capabilities of able-bodied individuals; those which enable people with disabilities to accomplish movements they cannot do independently; and therapeutic exoskeletons used for rehabilitation to help, resist or perturb the user’s movements [3]. Traditionally, it is the experience of the physicians that assesses the training during rehabilitation processes with these devices. In addition to that, kinematics and force measurements have gained prominence as objective tools to support clinical evaluation. While these physical metrics are essential, relying solely on them leaves an evaluation gap regarding the user’s cognitive load [4]. In fact, although exoskeletons continue to evolve in terms of mechanical sophistication and effectiveness, their widespread and long-term adoption remains limited. The major obstacle hindering their daily use is no longer just physical, but regards the high mental workload involved [5]. Even if there is a plurality of definitions about what mental workload is, we opt for the one related to the field of human–robot interaction, including the wearable assistive devices, as the exoskeletons. That is why this cognitive effort could be described as “the amount of demand placed on the user by the system” [6]. Walking is typically an automatic activity for able-bodied individuals, while operating a powered exoskeleton necessitates a significant concentration level, requiring focus at each step [7]. This heightened cognitive demand restricts the user from navigating uneven surfaces and hinders the ability to engage in concurrent activities, such as holding a conversation or processing complex visual information from the surroundings [6,7,8]. Within this context, visuospatial attention is a critical component during exoskeleton-assisted locomotion [9,10]. The high cognitive demand of operating an exoskeleton can saturate the user’s limited neural resources, impairing their ability to scan the environment and increasing fall risks [11,12]. Under these conditions of cognitive overload, a phenomenon known as ”inattentional blindness” [13] is often triggered: the brain becomes so absorbed by the primary task that it ceases to consciously process visual information. Consequently, users may physically direct their gaze straight to an obstacle or hazard without actually perceiving or registering its presence. Because of this discrepancy between mechanical gaze orientation and actual conscious perception, relying on metrics that only track eye direction is insufficient to quantify true cognitive effort. A robust assessment of true mental workload, therefore, requires the integration of active visuospatial paradigms capable of measuring actual attentional engagement in parallel with physical performance. A notable example that successfully addresses the issue of inattentional blindness through target–distractor differentiation is the Standing and Walking Visual Attention Field (SWAVF) task [9]. This task has been developed to evaluate covert visuospatial attention during locomotion. It requires users to maintain a forward gaze while actively scanning the lower peripheral visual field. During the walk, participants are prompted to identify a specific target hidden among several distractors (e.g., differentiating a yellow light from multiple green lights) and must make a deliberate behavioral response to indicate the target’s location. By forcing this active target–distractor differentiation and a physical reaction to the stimuli, the SWAVF paradigm guarantees that the visual information was successfully and consciously processed. However, while this paradigm is highly effective, it was originally validated exclusively during seated and unassisted walking, without the inclusion of any robotic device. Furthermore, its physical setup is inherently confined to treadmill-based walking. Together, these factors restrict the evaluation to controlled laboratory setups and prevent a truly ecological assessment of human–robot interaction in overground scenarios. This specific gap is reflected in the broader literature, where only a small fraction of cognitive assessment studies (approximately 20%) focus on exoskeletons compared to prostheses [5]. To overcome traditional limitations, recent studies have explored advanced technologies to assess human–robot interaction. However, current protocols evaluate biomechanical and cognitive metrics separately rather than within a unified framework. Eye-tracking and pupillometry successfully evaluate the cognitive burden of prosthetic hands [14] and lower-limb exoskeletons [15,16]. However, these studies perform measurements independently and lack integration with secondary tasks. Augmented and Virtual Reality (AR/VR) paradigms provide controlled cognitive stimuli. However, the previous literature restricts these applications strictly to static industrial scenarios or upper-limb rehabilitation [17,18,19]. International benchmarking initiatives, such as EUROBENCH, standardize the collection of biomechanical and physiological data during exoskeleton use [20]. Nevertheless, these frameworks operate in laboratory setups and lack ecological environments. Consequently, existing methodologies fail to simultaneously assess physical performance and cognitive workload under realistic conditions [21]. To the best of our knowledge, no existing research has combined dual-task paradigms, extended reality (XR), kinematics, and eye-tracking measurements to evaluate exoskeleton-assisted locomotion. To address this literature gap, this study presents the metrological validation of a novel measurement protocol that aims to objectively assess exoskeleton-assisted locomotion. The proposed framework integrates an IMU-based motion capture system (Xsens MTw Awinda) with wearable eye-tracking technology (Pupil Neon) embedded within a Mixed Reality (XR) headset (Meta Quest 3). This configuration enables users to perform overground walking while navigating a holographic environment, facilitating an ecological evaluation of motor progress while actively stimulating visuospatial attention. Specifically, the concurrent cognitive task requires participants to visually identify, fixate upon, and verbally report a green number appearing in their peripheral field of view. To intentionally increase the cognitive demand, participants were instructed to report the number in English, which was not their primary language. Through this setup, the main objective of this work is to demonstrate that the system is sensitive enough to monitor the training process. We focus on capturing both kinematic progress and the associated cognitive demand as users adapt to the device, establishing a practical framework for assessing how humans and robotic devices work together.

## 2. Materials and Methods

**Figure 1:**
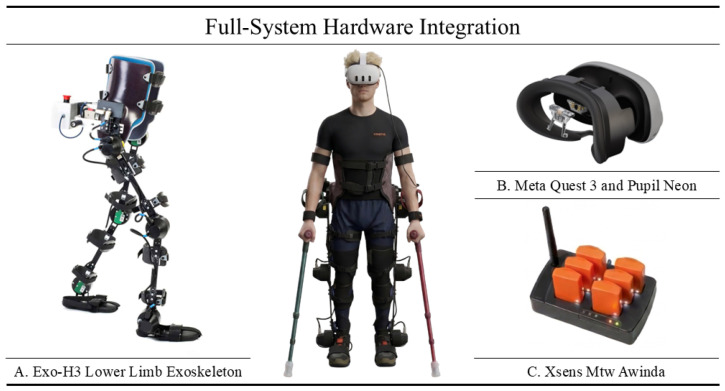
Overview of the integrated multi-modal experimental setup. The complete on-body setup worn by a user includes: (**A**) Exo-H3 lower limb exoskeleton; (**B**) Meta Quest 3 headset with Pupil Neon eye-tracking; and (**C**) Xsens MTw Awinda inertial sensors.

The experimental framework (Figure 1) evaluates motor and cognitive adaptation during exoskeleton-assisted walking through a novel dual-task paradigm. Thirty healthy participants performed overground walking with a lower-limb exoskeleton (Exo-H3) while simultaneously engaging in a mixed-reality visual search task (Meta Quest 3). Kinematic stability (Xsens IMUs) and cognitive workload (Pupil Neon eye-tracking) were continuously monitored across repeated training sessions to objectively quantify the learning process and the human–robot interaction.

### 2.1. Materials

The Exo-H3 lower-limb exoskeleton (Technaid S.L., Madrid, Spain) developed in collaboration with the CSIC Neurorehabilitation group, has been used to safely replicate a rehabilitation scenario in this study with healthy subjects. This device provides six degrees of freedom in the sagittal plane, assisting the flexion and extension of the hips, knees, and ankles for both legs.

The wireless MTw Awinda system (Xsens Technologies B.V., Enschede, The Netherlands) has been adopted to record users’ movement and accurately extract the full-body kinematics through the MVN Analyze software 2023.2 [22]. This setup uses 17 synchronized inertial measurement units (IMUs) operating at a sampling frequency of up to 60 Hz. This configuration has been adopted because already been validated by comparison against gold standard instrumentation for gait analysis [23,24,25].

Furthermore, the system integrates a Meta Quest 3 (Meta Platforms, Inc., Menlo Park, CA, USA) to administer the cognitive task in Mixed Reality (MR) and Pupil Neon glasses (Pupil Labs GmbH, Berlin, Germany) to monitor visual attention and pupillometry. The eye-tracker glasses have been validated in the work of Pfeffer, T. et al. [26] for cognitive measurement. The headset’s RGB cameras and depth sensors allow for the overlay of virtual elements onto the physical world, ensuring safe navigation. Moreover, the eye tracker streams real-time gaze data to the Meta headset via the Neon XR Core Package.

Finally, the dual-task application was developed using Unity v2022.3.62f2 (Unity Technologies, San Francisco, CA, USA). All the data analysis has been carried out using Matlab R2024B (The MathWorks, Inc., Natick, MA, USA) and executed on a Dell XPS 9520 with a Intel Core i7-12700H @ 2.30 GHz processor.

### 2.2. Dual-Task Design

The cognitive activity designed to measure visuospatial attention was developed using the Standing and Walking Visual Attention Field (SWAVF) [9] task as a foundational concept. The original SWAVF task relies on a physical LED platform, involves walking on a treadmill, and forces participants to maintain their gaze on a fixed central point. Our custom version, instead, projects the task directly through the headset, allowing the subject to view the real world and freely move. This design eliminates the need to walk on a treadmill or to fixate the gaze on a specific location, thereby enabling natural visual exploration.

The trial begins by verifying the eye-tracker communication with the Meta headset. Participants observe two virtual white spheres reflecting their real-time gaze. The experiment proceeds upon the vocal confirmation. After this phase, the subject is instructed to perform specific head movements (nodding and shaking the head). This action generates a distinct kinematic signature across the data streams, serving as a backup mechanism for the offline temporal synchronization of all the involved sensors.

**Figure 2:**
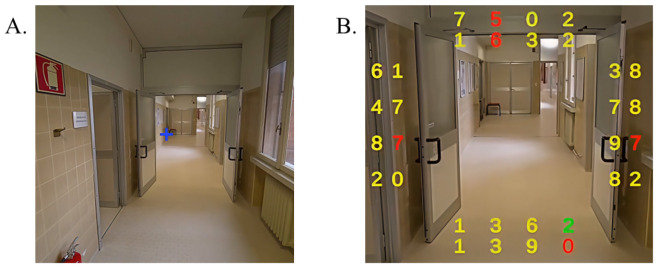
First-person perspective of the custom visuospatial attention task projected via the mixed-reality headset. (**A**) The trial cycle initiates with the presentation of a central blue cross. (**B**) Subsequently, a grid of colored numbers is displayed in the user’s peripheral vision over the real-world environment. Participants must visually locate and verbally identify the single green target number (e.g., ’2’ in the bottom right) embedded among yellow numbers and red distractors.

Then the actual cognitive task (Figure 2) can formally start.

The task operates cyclically, with trials repeating at randomized intervals of 2 to 4 s. Each cycle begins with a central blue cross (Figure 2A), immediately followed by a number grid in the user’s peripheral vision (Figure 2B). This grid consists of yellow numbers, red distractors, and a single green target. Participants must visually locate the green target and read it aloud as fast as possible in English; using a non-native language serves to slightly elevate the cognitive demand. Furthermore, this verbal response replaces the manual clickers used in the original SWAVF task [9] to prevent inattentional blindness. This ensures conscious visual processing while avoiding any physical constraints. Since operating the exoskeleton requires the use of crutches for stability, holding and operating manual clickers would be highly impractical.

During the task, the application continuously logs the spatial positions and the exact appearance and disappearance timestamps of the target (green) and distractor (red) numbers. Additionally, the system records the user’s real-time gaze coordinates within the virtual environment, their head position, and continuous audio of their vocal responses.

### 2.3. Methods

#### 2.3.1. Pupil Neon & Meta Quest 3 Gaze Calibration

A gaze calibration is needed to confirm that the user’s gaze actually lands on the target number that is then verbally reported. In fact, the raw gaze data originates from the Pupil Neon sensor’s local reference frame, while the holographic numbers exist within the Meta headset’s 3D virtual coordinate system. Furthermore, the physical adapter holding the eye tracker can vary slightly in position and orientation depending on the user’s facial anatomy. Therefore, a spatial transformation is required for each user to align these two distinct reference frames.

The calibration process uses the PL_Calibration scene, an official, pre-built tool provided by Pupil Labs as part of their Neon XR Unity package. The VR headset sequentially renders a series of visual markers at predefined, fixed 3D coordinates within the virtual environment. As the user focuses on each marker, the system continuously pairs the raw gaze vectors with the exact 3D position of the virtual target. Once a sufficient number of samples is collected, the software employs an iterative Kabsch algorithm to compute the optimal rigid body transformation. Specifically, it calculates the translation and rotation matrices that minimize the root-mean-square deviation (RMSD) between the projected gaze points and the actual target coordinates. The transformation matrix is then saved as a configuration file, which can be applied globally to convert real-time, raw gaze data into VR world-space rays.

#### 2.3.2. Pupil Neon & Meta Quest 3 Synchronization

Eye-tracking data were simultaneously recorded via two parallel pipelines to ensure redundancy and maximize temporal resolution. The Unity application (via the Pupil Neon XR Core package) recorded basic parameters, including raw gaze position and pupil diameter, at approximately 80 Hz. Concurrently, the Pupil Labs Companion device performed a high-frequency local recording at 200 Hz, extracting both basic parameters and advanced metrics (blinks, saccades, and fixations).

Because these asynchronous streams operated at different frequencies, we synchronized them using the raw horizontal gaze position ($X_{gaze}$). The low-frequency Quest signal was first linearly upsampled to match the Companion’s 200 Hz timestamps. We then applied cross-correlation to estimate an initial time lag. To precisely align the streams, this initial delay seeded an iterative optimization algorithm. The optimal time delay (Δt) was computed by minimizing a mean squared error (MSE) cost function, J(Δt):

(1) $$J\left(\Delta t\right)=\frac{1}{K}\sum_{k=1}^{K}{\left(x_{pupil}\left(t_{k}\right)-x_{quest}\left(t_{k}-\Delta t\right)\right)}^{2}$$

where *K* represents the total number of valid samples. Applying the optimized offset ($\Delta T^{*}$) globally synchronized all high-frequency Companion metrics with the Meta Quest virtual environment.

Raw gaze position served as the primary synchronization signal because both systems log the identical sensor stream, differing only by network delay. To ensure system robustness against potential network interruptions, a secondary kinematic fallback strategy was implemented. This backup procedure synchronized the streams by aligning the physically distinct inertial signals (IMUs) recorded independently by the Meta Quest 3 and the Pupil Neon.

The spatial synchronization residual was quantified as the root mean square error (RMSE) between the two signals evaluated at the optimal temporal offset $\Delta T^{*}$: $\sigma_{x}=\sqrt{J(\Delta T^{*})}$. Pooled across all synchronized recordings, the spatial residual was $\sigma_{x}=37.9$ px ($\approx 2.0^{\circ }$ of visual angle, Meta Quest 3 display: 2064 × 2208 px/eye, horizontal FoV $\approx 110^{{}^{\circ}}$).

#### 2.3.3. Event Detection & Step Segmentation

Stationary and turning intervals were excluded by tracking the knee sagittal flexion-extension angle, retaining only steady-state walking segments. These data were segmented into stance and swing phases using the algorithm proposed by Grimmer et al. [27] to extract Heel Strike (HS) and Toe Off (TO) events. The algorithm first identifies local maxima in the knee sagittal angle. For a time series $y(kT_{s})$ with sampling time $T_{s}$ and frame index *k*, a local maximum is defined as:

(2) $$y\left(\left(k-1\right)T_{s}\right)<y\left(kT_{s}\right)>y\left(\left(k+1\right)T_{s}\right)$$

False positives are filtered using a minimum expected step duration (2 s) and a minimum angular amplitude of 30°, partitioning the continuous cycle into distinct peak-to-peak windows. Within each window, HS and TO events are identified via zero-crossings in the shank angular velocity. HS is defined as the first negative-to-positive transition sustained for at least 10 sampling intervals, occurring no earlier than 200 ms after the window’s onset. Conversely, TO is identified as the first positive-to-negative transition sustained for 10 intervals, constrained to occur after 60% of the window duration has elapsed. These temporal and kinematic constraints effectively account for the altered gait patterns induced by the exoskeleton, while allowing for manual refinement in cases of excessive signal noise.

#### 2.3.4. Kinematic Metrics

To prevent the high dimensionality and multiple-testing correction issues that can reduce statistical power in gait analysis, a targeted selection strategy was adopted. The analysis focused on three complementary metrics to evaluate movement fluency and gait quality under dual-task cognitive conditions: Step Length Variability (SLV), Spectral Arc Length (SPARC), and the Coefficient of Multiple Correlation (CMC). SPARC and CMC were computed using the sagittal angular velocity of the shank. This approach aligns with the existing literature using minimal sensor setups [28,29,30]. Foot and ankle signals were explicitly excluded due to ground impact artifacts. Moreover, many exoskeletons lack active ankle actuation. During the swing phase, the shank acts as a pendulum. This dynamic provides an optimal signal-to-noise ratio for waveform-based algorithms.

Step Length Variability (SLV) serves as a sensitive indicator of motor stability, spatial asymmetry, and potential fall risk during exoskeleton-assisted walking [31,32]. It captures step-to-step fluctuations and is quantified as the coefficient of variation (CV). Defining μ as the mean step length and σ as the standard deviation of the step length, the CV is calculated as:

(3) $$SLV=CV=\frac{\sigma }{\mu }$$

A lower SLV indicates reduced variability and a more stable, controlled gait, whereas a higher SLV reflects elevated step-to-step instability [33].

To evaluate motion smoothness, we computed the Spectral Arc Length (SPARC) of the shank angular velocity. SPARC is a robust metric derived from the signal’s frequency spectrum, which compensates for variations in walking speed. It is defined as the negative arc lenght of the normalized Fourier spectrum amplitude, $\hat{A}\left(\omega \right)$:

(4) $$SPARC=-\int_{0}^{\omega_{c}}\sqrt{{\left(\frac{1}{\omega_{c}}\right)}^{2}+{\left(\frac{d\hat{A}\left(\omega \right)}{d\omega }\right)}^{2}}d\omega$$

The cutoff frequency $\omega_{c}$ was determined following the standard adaptive methodology introduced by Balasubramanian et al. in [34]. In particular, $\omega_{c}$ is identified as the minimum frequency above which the normalized magnitude spectrum of the speed profile continuously remains below a given threshold, set to 0.05. Smoother movements concentrate the frequency spectrum at lower frequencies, yielding less negative SPARC values, while more irregular or jagged movements produce more negative values [35,36].

Finally, waveform similarity between repeated kinematic signals, specifically, normalized shank angular velocity profiles, was evaluated using the Coefficient of Multiple Correlation (CMC) [37]. This dimensionless measure integrates the effects of vertical offset, curve shape, and amplitude scaling. Defining *F* as the number of frames in a gait cycle, *N* as the number of cycles, $Y_{ij}$ as the value at frame *j* in cycle *i*, ${\bar{Y}}_{j}$ as the mean at frame *j* across all cycles, and $\bar{Y}$ as the grand mean of all values, the CMC is calculated as the square root of 1 minus the ratio of residual variance to total variance:

(5) $$CMC=\sqrt{1-\frac{\sum_{i=1}^{N}\sum_{j=1}^{F}{\left(Y_{ij}-{\bar{Y}}_{j}\right)}^{2}}{\sum_{i=1}^{N}\sum_{j=1}^{F}{\left(Y_{ij}-\bar{Y}\right)}^{2}}}$$

The CMC quantifies reliability between sessions, with values approaching 1 indicating waveform overlap after compensating for systematic biases.

#### 2.3.5. Cognitive Metrics

To quantify the cognitive demands of the dual-task conditions, we analyzed pupil diameter data continuously recorded by the integrated eye-tracking system. Raw bilateral pupil signals were preprocessed by detecting and removing blink artifacts, applying linear interpolation to fill the resulting data gaps, and averaging the left and right pupil diameters to obtain a unified continuous time series. Cognitive effort was then evaluated using two complementary indices: normalized continuous pupil dilation and the Task-Evoked Pupillary Response (TEPR).

First, to assess sustained cognitive workload, we analyzed the continuous pupil dilation. In cognitive pupillometry, the continuous (or tonic) pupil diameter serves as a reliable physiological proxy for autonomic nervous system arousal, reflecting the sustained mental effort required to engage in a prolonged activity [38,39]. To account for inherent inter-subject variability in resting pupil size, these raw measurements were z-score normalized for each participant across all experimental conditions.

Second, to capture the acute mental effort associated with processing individual stimuli, we calculated the Task-Evoked Pupillary Response (TEPR) [40,41]. This metric evaluates the phasic pupillary dilation triggered by a specific cognitive event, in our case the cognitive task of identifying the target stimulus. The TEPR was extracted by time-locking the preprocessed pupil signal to the exact onset of each target green number (t = 0). A pre-stimulus baseline ($D_{baseline}$) was established using the mean pupil diameter over a 1-s window immediately preceding the stimulus appearance (from t=−1 s to t=0 s). Subsequently, the peak pupil diameter ($D_{max}$) was identified within a 2-s post-stimulus window (from t=0 s to t=2 s). The TEPR was then computed as the maximum proportional increase relative to the baseline, expressed through the following equation:

(6) $$TEPR=\frac{D_{max}-D_{baseline}}{D_{baseline}}$$

By isolating this phasic response, this metric quantifies the immediate cognitive cost of identifying and processing the target, independently of the user’s overall tonic arousal.

### 2.4. Experimental Setup

The experimental protocol included sessions with the exoskeleton alone (training), the VR headset alone (single-task test), and a combination of both (dual-task test).

The exoskeleton was operated in active mode using a position control paradigm; it provides full assistance over a pre-defined trajectory. The assistance and initiation of the walking cycles were manually activated and controlled by the experimenter. The walking speed of the exoskeleton was set during the preliminary familiarization session.

The 17 wireless IMUs were positioned on the participant’s body according to the standard Xsens MVN full body configuration [42]. In particular, the sensors were attached to the following body segments: head, sternum, pelvis, bilaterally on the shoulders, upper arms, forearms, hands, thighs, shanks, and feet.

To simulate a realistic walking scenario, all experiments were conducted in a standard corridor under ecological conditions. Specialized control equipment was not used for light control. Ambient luminance variability was minimized by relying on constant artificial illumination, with no exposure to direct natural light.

Data acquisition across all sensors and the Meta Quest 3 application was triggered simultaneously via a custom Python-based program (Python 3.11.4). Specifically, this program executed shell commands via TCP/IP protocols and proprietary libraries to remotely initiate the Unity application on the Meta Quest and start the eye-tracking recording on the Pupil Neon hardware. Concurrently, the interface triggered the Xsens motion capture recording locally on the host PC.

All devices were connected to the same local network to establish a shared temporal reference, and the synchronization between the Meta Quest and Pupil eye-tracking data streams was subsequently fine-tuned offline using cross-correlation and mathematical optimization.

### 2.5. Experimental Protocol

**Figure 3:**
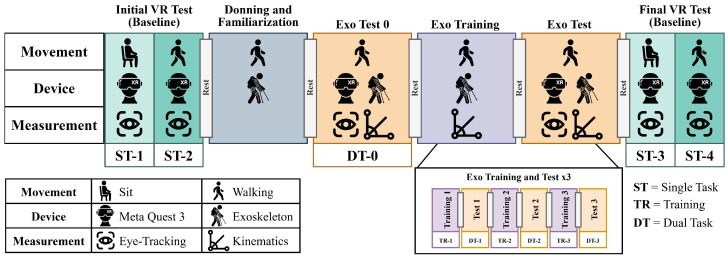
Schematic overview of the experimental protocol. The timeline illustrates the sequence of Single Task (ST) and Dual Task (DT) conditions. The protocol begins with initial XR assessments (ST-1, ST-2), followed by exoskeleton donning and a baseline dual-task test (DT-0). Participants then undergo three consecutive cycles of exoskeleton training (TR-1 to TR-3) and dual-task testing (DT-1 to DT-3), concluding with final XR assessments (ST-3, ST-4). The grid explicitly details the movements (sitting, walking), involved devices (Meta Quest 3, Exoskeleton), and the acquired data (Eye-Tracking, Kinematics) for each corresponding phase. Interleaved “Rest” blocks denote pauses between tasks.

To validate the protocol, 30 healthy subjects (15 male and 15 females) have voluntarily participated in the experiment following the protocol in Figure 3. The recruited participants had an age of 26.1 ± 3.6 years, ranging from 20 to 40 years, an height of 171.1 ± 7.8 cm, ranging from 160 to 185 cm, and a body mass of 67.2 ± 10.6 kg, ranging from 50 to 80 kg. The study was performed according to the guidelines of the Ethics Committee for University Research of the University of Brescia, identified with the code 45/2025.

The experimental protocol begins with an Initial VR Test (Baseline). For each trial involving the headset, the task concluded once the subject was presented with 20 numbers within the application, resulting in a duration of approximately 2.5 min per session. During this initial baseline phase, the subject wears a Meta Quest 3 headset and performs tasks in both a seated (ST-1) and walking (ST-2) position. Through the headset, only Eye-Tracking data is recorded to establish an attentional baseline. To mitigate the effects of physical and cognitive fatigue, custom-duration rest periods are integrated between all consecutive testing and training phases.

Then, a 15-min session is dedicated to donning the exoskeleton, and a brief familiarization with the exoskeleton is performed to adjust the device’s parameters to the individual’s biomechanical needs.

Following this adaptation and an additional rest period, Exo Test 0 (DT-0) is conducted. The participant walks while simultaneously wearing the exoskeleton and the Meta Quest 3. During this initial stage of combined use, both Kinematics and Eye-Tracking data are acquired simultaneously.

After this initial measurement, the protocol employs an iterative loop to evaluate the progression of the user’s motor adaptation. This loop consists of an Exo Training session (TR), where the subject walks with only the exoskeleton while kinematic data is recorded, followed by an Exo Test (DT) phase. The Exo Test replicates the exact conditions of Exo Test 0, acquiring both kinematic and ocular data while the subject walks with the exoskeleton and interacts with the Meta Quest 3. As detailed in the diagram (Exo Training and Test ×3), this combined block is repeated three consecutive times in an alternating fashion, with rest periods interspersed between each individual training and test session to capture progressive adaptation.

Finally, the protocol concludes with a Final VR Test (Baseline). Mirroring the initial phase, the participant performs seated and walking tasks wearing only the Meta Quest 3 headset (monitored via eye-tracking). This procedure is designed to capture concluding cognitive metrics and account for any potential fatigue accumulated throughout the entire experiment.

Rest periods between experimental blocks were defined ad hoc based on participants’ verbal feedback rather than fixed time intervals. All 30 participants successfully completed the protocol, and no subjects or individual trials were excluded from the analysis.

The sequence of the experimental blocks was fixed to simulate a progressive training program. This protocol was condensed into a shorter timeframe, as healthy participants require less training to adapt to an exoskeleton [43]. In this protocol, the XR sessions act as punctual tests to assess the user’s current position on the learning curve.

### 2.6. Statistical Analysis

#### 2.6.1. Statistical Power and Sample Size

A sample size of N=30 was chosen to ensure robust statistical power for the Linear Mixed-Effects Models (LMM). This aligns with established standards in human–robot interaction and biomechanics. Recent studies evaluating lower-limb exoskeletons typically rely on 20 to 30 healthy participants [44,45,46,47].

To justify this choice, we conducted a Monte Carlo sensitivity analysis simulating 1000 iterations for our specific experimental design.

Model parameters, including variance components and a conservative effect size (Cohen’s d≈0.80), were estimated from existing literature and pilot observations. The simulation confirmed that testing 30 participants yields a statistical power exceeding 95% (α=0.05). Consequently, this sample size reliably detects significant within-subject adaptations while minimizing Type I and Type II errors.

#### 2.6.2. Statistical Evaluation of the Experimental Metrics

Prior to the main statistical analyses, the Lilliefors test was conducted to evaluate the normality of the data distributions.

For the kinematic parameters (SLV, SPARC and CMC), the null hypothesis of normality was not rejected in any of the experimental conditions, justifying the use of parametric statistics. Accordingly, Linear Mixed Models (LMMs) were implemented. The LMMs included the experimental *Session* and the *Task* modality (Training vs. Dual Task) as interacting fixed effects, while the *Subject* was modeled as a random intercept to account for individual baseline differences (model formulation: Metric ∼ Session * Task + (1|Subject)).

To specifically assess how training impacted performance under cognitive load, we targeted our analysis on the dual-task conditions. Accordingly, an omnibus Analysis of Variance (ANOVA) was performed on the Dual Task conditions. For any significant effects, subsequent post-hoc pairwise comparisons using paired *t*-tests with Bonferroni correction were conducted.

For the cognitive workload analysis based on pupillometry, the Lilliefors test revealed that not all conditions met the assumption of normality. Consequently, a non-parametric statistical approach was adopted. Global differences in the normalized continuous pupil dilation across the experimental conditions were evaluated using the Friedman test. When significant main effects were detected, post-hoc pairwise comparisons were conducted using the Wilcoxon signed-rank test to identify specific condition-level differences.

Finally, to mathematically model the cognitive adaptation over time, LMMs were fitted for the Task-Evoked Pupillary Response (TEPR) isolated during the exoskeleton-assisted sessions. In these specific models, the longitudinal *Session* was treated as a fixed effect to capture the time-dependent learning trend, with the *Subject* included as a random intercept (model formulation: Metric ∼ Session + (1|Subject)).

## 3. Results

### 3.1. Kinematics Results

Table 1 details the statistical evaluation of the three extracted kinematic metrics, summarizing the outcomes of both the LMMs and the subsequent post-hoc comparisons across the Dual Task conditions.

**Figure 4:**
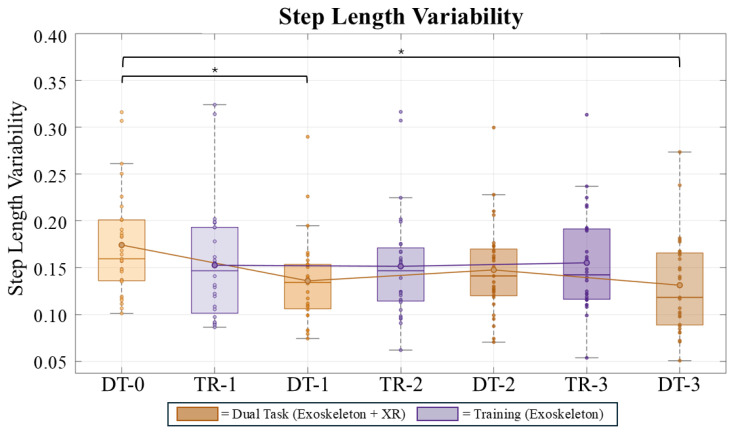
Step Length Variability (SLV) across the sequence of training (TR) and dual-task (DT) assessment sessions. Orange boxes denote the Dual-Task condition (Exoskeleton + XR Headset), and purple boxes denote the Training condition (Exoskeleton only). For both conditions, the transition from lighter to darker shades represents the chronological progression of the sessions over time. Solid lines connect the mean values across conditions. Statistically significant differences between sessions are indicated by black brackets with asterisks (* *p* < 0.05).

The LMM analysis of the Step Length Variability (SLV) (Figure 4) indicated no significant baseline difference between the conditions (*p* = 0.085). A highly significant main effect of Session was found (*p* < 0.001), demonstrating a decrease in gait variability over time. Furthermore, a significant Session × Task interaction was detected (*p* = 0.020), indicating that the reduction in variability observed in the Dual Task condition was absent in the Training condition. The RM-ANOVA confirmed a significant main effect of Condition, representing a large effect size ($\eta_{p}^{2}=0.267$). Post-hoc comparisons on Dual-Task sessions indicated that the initial assessment DT-0 was characterized by significantly higher spatial variability compared to condition DT-1 (*p* = 0.006, d=0.711, indicating a medium-to-large effect) and condition DT-3 (*p* = 0.007, d=0.701, also a medium-to-large effect), although the difference with condition DT-2 did not reach statistical significance (*p* = 0.107). No significant differences were observed among the subsequent assessments (DT-1, DT-2, and DT-3).

**Figure 5:**
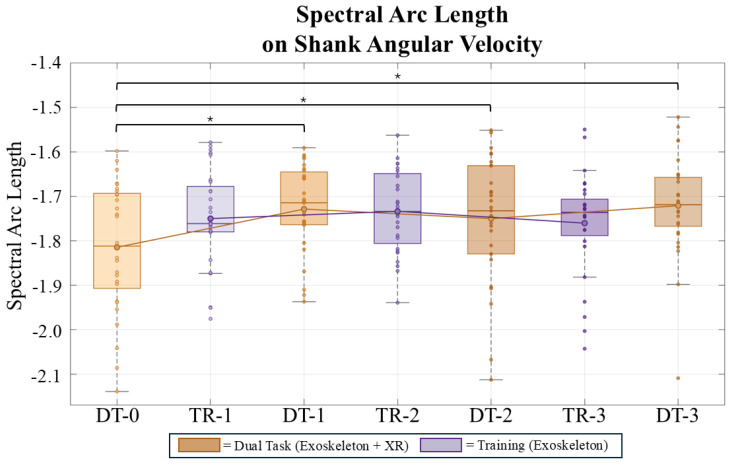
Spectral Arc Length (SPARC) on the Shank Angular Velocity across the sequence of training (TR) and dual-task (DT) assessment sessions. Orange boxes denote the Dual-Task condition (Exoskeleton + XR Headset), and purple boxes denote the Training condition (Exoskeleton only). For both conditions, the transition from lighter to darker shades represents the chronological progression of the sessions over time. Solid lines connect the mean values across conditions. Statistically significant differences between sessions are indicated by black brackets with asterisks (* *p* < 0.05).

The LMM analysis of the Spectral Arc Length (SPARC, Figure 5), where less negative values indicate a smoother gait, the model revealed a significant main effect of Task at baseline (*p* = 0.023), confirming that subjects initially exhibited a significantly smoother movement during the Training. A significant main effect of Session was also observed (*p* < 0.001), showing a clear improvement in smoothness over time during the Dual Task condition. Crucially, a significant Session × Task interaction was found (*p* = 0.019), demonstrating that while smoothness significantly improved over the sessions in the Dual Task, it remained relatively stable in the ST, leading to a progressive convergence of the two conditions. Additionally, a significant main effect of Condition was highlighted by the RM-ANOVA, capturing a large effect size ($\eta_{p}^{2}=0.225$). Post-hoc comparison on Dual-Task sessions revealed that movement smoothness was significantly lower at condition DT-0 compared to condition DT-1 (*p* = 0.002, exhibiting a large effect size, d=0.812), condition DT-2 (*p* = 0.037), and condition DT-3 (*p* = 0.033, which showed a medium effect size, d=0.582). No significant differences were observed among the subsequent assessments (DT-1, DT-2, and DT-3).

**Figure 6:**
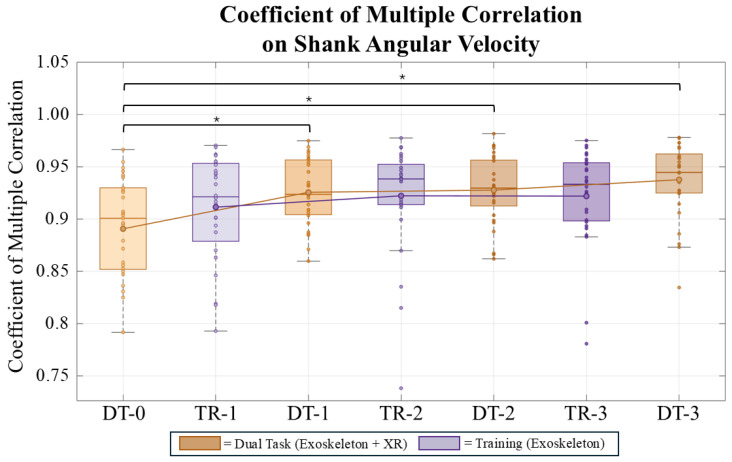
Coefficient of Multiple Correlation (CMC) on the Shank Angular Velocity across the sequence of training (TR) and dual-task (DT) assessment sessions. Orange boxes denote the Dual-Task condition (Exoskeleton + XR Headset), and purple boxes denote the Training condition (Exoskeleton only). For both conditions, the transition from lighter to darker shades represents the chronological progression of the sessions over time. Solid lines connect the mean values across conditions. Statistically significant differences between sessions are indicated by black brackets with asterisks (* *p* < 0.05).

The LMM analysis on the Coefficient of Multiple Correlation (CMC, Figure 6) of the shank angular velocity demonstrated a highly significant positive main effect of Session (*p* < 0.001), indicating that movement similarity significantly improved over time. The baseline difference between the Training and Dual Task was not statistically significant (*p* = 0.147), and the Session × Task interaction only showed a trend without reaching statistical significance (*p* = 0.073), suggesting that the rate of improvement was not structurally different between the two conditions. In contrast, the RM-ANOVA revealed a prominent main effect of Condition with a remarkably large effect size ($\eta_{p}^{2}=0.439$). Post-hoc analysis demonstrated that the initial consistency in condition DT-0 was significantly lower than in all subsequent Dual-Task sessions (*p* < 0.001 for all comparisons against conditions DT-1, DT-2 and DT-3). These pairwise differences manifested as exceptionally large effect sizes for both DT-1 (d=1.093) and DT-3 (d=1.014). Consistent with the other results, the CMC values plateaued after the initial assessment, with no significant differences found between conditions DT-1, DT-2, and DT-3 (all *p* > 0.05).

Taken together, these analyses suggest a nonlinear adaptation during the Dual Task, featuring rapid initial improvements. Subsequently, the data hints at a potential kinematic plateau across the remaining evaluation phases.

### 3.2. Cognitive Results

Table 2 summarizes the statistical outcomes for the two evaluated cognitive metrics: normalized continuous pupil dilation and the Task-Evoked Pupillary Response (TEPR). Differences in continuous pupil dilation across the experimental conditions were analyzed using a non-parametric Friedman test, followed by Wilcoxon signed-rank tests for post-hoc pairwise comparisons. Conversely, the TEPR during the exoskeleton-assisted Dual-Task sessions was analyzed by fitting Linear Mixed-Effects Models (LMM).

**Figure 7:**
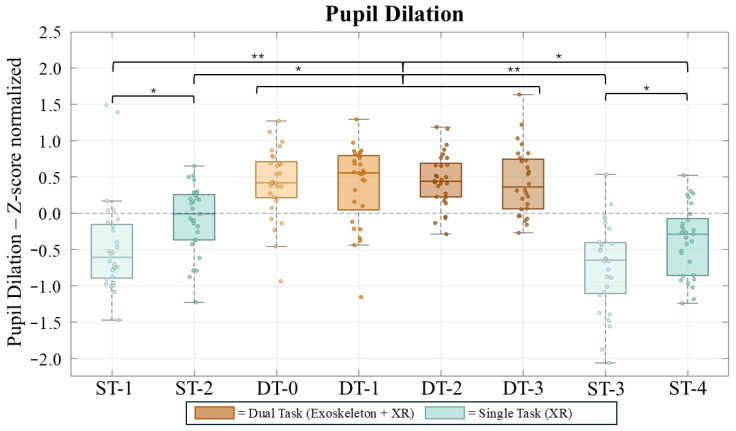
Z-score normalized pupil dilation across Single Task (ST, light blue) and Dual Task (DT, orange) experimental conditions. In the ST condition, two shades of blue are used to differentiate between the sitting and walking tasks. In the DT condition, orange boxes are used, where the transition from lighter to darker shades represents the chronological progression of the sessions over time. Statistically significant differences between sessions are indicated by black brackets with asterisks (*p* < 0.05 * and *p* < 0.001 **).

The Z-score normalized pupil dilation (Figure 7) was analyzed across eight experimental phases, comprising four Single Task (ST-1 to ST-4) and four Dual Task (DT-0 to DT-3) conditions. A non-parametric Friedman test revealed a significant main effect of the experimental condition on pupil dilation (p<0.001), reflecting a moderate-to-large effect size (W=0.431). Post-hoc pairwise comparisons using the Wilcoxon signed-rank test showed that normalized pupil dilation during all DT phases was significantly higher compared to both the seated ST phases (ST-1 and ST-3; p<0.001, with a large effect size for ST-1 vs. DT-0, r=0.803) and the walking ST phases (ST-2 and ST-4; p<0.05). No significant differences were found when comparing the four DT blocks with each other (*p*-values ranging from 0.681 to 0.927). Conversely, statistically significant variations were observed within the Single Task conditions, specifically between ST-1 and ST-2 (p=0.0215, unveiling a medium-to-large effect size, r=0.498) and between ST-3 and ST-4 (p=0.0342), which correspond to the seated (ST-1, ST-3) and walking (ST-2, ST-4) task modalities.

**Figure 8:**
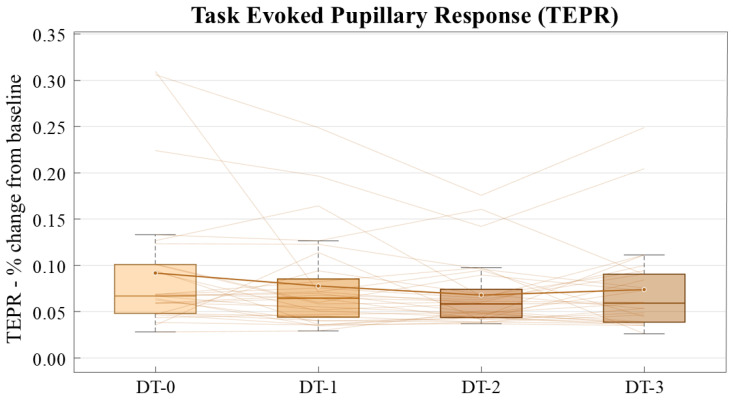
Task-Evoked Pupillary Response (TEPR), expressed as the percentage change from baseline, across the four DT sessions. The transition from lighter to darker shades represents the chronological progression of the sessions over time Thin faded lines illustrate individual participant trends. The boxplots represent the data distribution for each session, with the solid line connecting the group means.

The Task-Evoked Pupillary Response (TEPR, Figure 8), expressed as the percentage change from baseline, was analyzed across the four repeated Dual Task (DT) sessions. A Linear Mixed Model (LMM) was employed to assess the fixed effect of the ’Session’, accounting for inter-subject variability. The LMM analysis revealed a statistically significant, small negative effect of the session progression on the TEPR amplitude (β=−0.0058, p=0.0255), with an estimated intercept of 0.0928 (p<0.001), representing an initial average TEPR of approximately 9.28% above baseline.

Ultimately, these results hint that while the overall tonic dilation remained consistently high during complex tasks, the amplitude of the phasic pupillary response decreased linearly, suggesting an adaptation to the Dual Task over time.

## 4. Discussion

The primary objective of this study was to validate a protocol for quantifying motor and cognitive adaptation during human-exoskeleton interaction. Before deploying such a complex architecture in clinical populations, it was essential to verify its reliability on a controlled cohort. The findings confirm the robustness of the proposed protocol. The integrated system successfully captured the dynamics of dual-task interference, tracking the transition from initial motor disruption to the eventual motor learning and stabilization.

### 4.1. Kinematics

Initially, the kinematic analysis revealed that the first assessment block (DT-0) was characterized by the lowest movement smoothness (SPARC) and the highest SLV. Since kinematic data were not recorded during the preceding familiarization phase, these initial baseline values likely reflect a combination of cognitive-motor interference and the overall novelty of the complex experimental setup (i.e., walking with the exoskeleton while engaging in the XR environment for the first time). However, a clear and rapid habituation effect was observed immediately after this first session. The significant improvements in smoothness and variability starting from TR-1 and DT-1 suggest that users quickly adapted to the system. Interestingly, the macro-structure of the gait pattern, measured by kinematic consistency (CMC), was less impacted at baseline. This is likely because fundamental rhythmic locomotion is governed by lower-level Central Pattern Generators (CPGs), which are highly resilient to cognitive effort [48,49]. Crucially, our measurement architecture captured a counterintuitive trend: as subjects automated the cognitive demands, the Dual Task (DT) performances did not just recover but seemed to surpass the Training (TR) sessions. By the end of the protocol, the architecture quantified smoother movements, higher consistency, and lower variability in the Dual Task condition compared to the initial training baseline. From a metrological standpoint, the ability to track this continuous improvement, without instrumental drift over multiple sessions, supports the robustness of the selected metrics.

Furthermore, the intra-condition analysis (Repeated Measures ANOVA) on the longitudinal DT assessments highlighted the system’s accuracy in measuring the actual physical constraints of robotic assistance. The data revealed a non-linear motor adaptation: the most substantial biomechanical improvements occurred abruptly between the first and second Dual Task assessments (DT-1, DT-2), immediately followed by a statistically robust plateau. This rapid stabilization is both biologically and mechanically sound. First, the healthy participants in this study possess intact neural circuitry and high cognitive reserve, which enable extremely fast motor learning when facing new dual-task challenges. Second, and most importantly, the active exoskeleton inherently restricts the user’s movements by imposing predefined spatial trajectories and fixed assistance timings. The initial drop in performance simply reflects a brief user-robot desynchronization caused by cognitive distraction and by the novelty of the task. Once the subjects learned to “trust” the exoskeleton and stopped consciously fighting its assistance, they synchronized with the robot’s rhythm. Because the device fundamentally dictates the joint kinematics through fixed assistance, the performance metrics quickly reached a kinematic plateau. The fact that our sensing architecture accurately recorded this stabilization, without showing random noise or artifactual fluctuations in the later sessions, confirms its reliability. Ultimately, while these results successfully demonstrate the feasibility and sensitivity of the measurement framework in healthy subjects, they just suggest its potential utility for future clinical applications. Further validation involving neurologically impaired patients, longer sessions, and different exoskeleton models is necessary before clinical deployment.

### 4.2. Cognitive

The cognitive assessment via pupillometry provided insights into how users allocate mental resources during exoskeleton-assisted overground walking. The continuous (tonic) pupil dilation, a physiological proxy for sustained arousal and general mental effort [40], was significantly elevated during all Dual Task (DT) phases compared to Single Task (ST) conditions. This physiological distinction highlights the cognitive overhead required to coordinate robotic assistance while simultaneously scanning the peripheral environment. Notably, the tonic dilation remained consistently high and stable across the four DT blocks. This lack of habituation in baseline arousal aligns with existing literature on cognitive-motor interference, which suggests that dynamic postural control and locomotion (especially when using an assistive device) demand continuous executive functioning that does not rapidly automate in novice users [50,51].

In contrast, the Task-Evoked Pupillary Response (TEPR) successfully captured the acute, phasic mental effort associated with processing the target stimuli. While the initial TEPR amplitude indicated a high immediate cognitive cost (averaging a 9.28% increase above baseline), the Linear Mixed-Effects Model revealed a significant, progressive decrease in this phasic response across the sessions. This gradual reduction in TEPR amplitude points toward a decrease in the acute cognitive resources required for the task. As users became more familiar with the peripheral visual search task and color-coded numbers, their perceptual processing became more efficient, demanding fewer acute cognitive resources per stimulus [40].

These cognitive findings support the effectiveness of our measurement protocol. The integrated system successfully separated two distinct mental processes occurring at the same time. On one hand, the continuous pupil dilation remained stable, reflecting the constant effort required to walk safely with the exoskeleton. On the other hand, the TEPR progressively decreased, showing that the peripheral visual search task demanded decreasing acute cognitive effort over time. This hints at a potential familiarization effect.

Combining kinematic and cognitive data explains how users handle dual-task interference. The results suggest that motor performance and mental load are linked over time. The first dual-task block (DT-0) is the most challenging. In this phase, acute cognitive demand (TEPR) reaches its peak. At the same time, the walking pattern exhibits instability. Step variability increases, and movement smoothness drops. However, gait stabilizes rapidly in the following blocks (DT-1 to DT-3). During this motor recovery, sustained mental effort (continuous pupil dilation) stays consistently high. On the other hand, the acute cost of the visual task (TEPR) steadily decreases. This specific pattern points to a ’posture-first’ strategy [50]. Users constantly dedicate high mental resources to walk safely with the exoskeleton. Meanwhile, they quickly adapt to the visual game. This habituation frees up acute cognitive resources. As a result, users can quickly restore and maintain their walking stability.

## 5. Conclusions

This study demonstrated the sensitivity of the novel sensing framework designed to monitor human-exoskeleton interaction. The system captured the initial disrupted gait control and sustained pupil dilation, caused by the novelty of the task and cognitive interference. It also successfully tracked the subsequent non-linear motor learning trend. Specifically, the framework detected a mechanical performance plateau imposed by the exoskeleton. At the same time, it recorded the physiological adaptation, highlighted by TEPR attenuation. Together, these findings highlight the reliability of the proposed architecture for continuous, and ecological monitoring.

Designing the protocol to be as ecological as possible inherently prevented strict luminance control. However, environmental lighting was kept stable. Ambient light was restricted to constant, indirect artificial illumination, and the brightness of the virtual stimuli was fixed within the Unity application. Moreover, the short duration of the tests ensured that external lighting did not fluctuate. Consequently, while absolute pupillary baselines may be influenced by the ecological setting, relative comparisons across experimental conditions remain robust.

Despite the promising findings, certain limitations must be acknowledged. The sample size, though statistically powered for this framework, restricts the broader generalizability of the results. The protocol was tested exclusively on healthy individuals. These participants do not reflect the clinical target demographic, such as stroke or spinal cord injury survivors, who are expected to exhibit higher performance variability and highly individualized learning curves. The system was tested using a single commercial exoskeleton model. Different robotic devices employ distinct control algorithms and assistance levels. Consequently, the current protocol may require adjustments before serving as a standard across diverse assistive platforms.

Building on these findings, future research will expand in several key directions. We will investigate the rapid early adaptation phase with higher temporal resolution by segmenting the initial dual-task block into smaller analytical epochs. We aim to identify subject-specific learning strategies by exploring behavioral clusters within the cohort. In this case, we will look for a correlation between the investigated metrics and the participants’ anthropometric information as well as their baseline physical activity and sex. The analytical framework will be enriched with additional metrics, such as fixation durations and vocal reaction times, to establish direct statistical correlations between kinematic improvements and cognitive engagement. A dedicated longitudinal study will isolate the exoskeleton learning curve, comparing purely motor training (Single Task) against concurrent training (Dual Task). This will thoroughly test the hypothesis that introducing a cognitive challenge, despite causing lower initial performance, may actually accelerate motor learning and ultimately produce a superior gait pattern. Finally, when transferring this framework to neurological populations, future designs must optimize the temporal scheduling between physical training and dual-task assessments to carefully manage patient fatigue while accurately tracking their distinct motor rehabilitation trajectories. 

## Figures and Tables

**Table 1:** Summary of the statistical analysis including Linear Mixed-Effects Models (LMM) for longitudinal predictive trends and Repeated Measures ANOVA for condition-based motor adaptation.

Statistical Test	Effect/Comparison	Test Statistic & Effect Size	*p*-Value
**Step Length Variability**			
LMM	Main Effect: Session	F(1,206)=15.15, β=−0.586	<0.001
LMM	Interaction: Session × Task	F(1,206)=4.90, β=0.626	0.020
RM-ANOVA	Main Effect: Condition	F(3,87)=9.62, $\eta_{p}^{2}=0.286$	<0.001
Post-Hoc (Bonferroni)	DT-0 vs. DT-1	d=0.788, 95%CI[1.194,7.642]	0.004
Post-Hoc (Bonferroni)	DT-0 vs. DT-3	d=0.698, 95%CI[0.778,8.040]	0.011
Post-Hoc (Bonferroni)	DT-1 vs. DT-2	d=−0.465, 95%CI[−3.544,0.376]	0.174
**SPARC V_SHANK**			
LMM	Main Effect: Session	F(1,206)=14.09, β=0.013	<0.001
LMM	Interaction: Session × Task	F(1,206)=5.45, β=−0.015	0.019
RM-ANOVA	Main Effect: Condition	F(3,87)=7.30, $\eta_{p}^{2}=0.226$	<0.001
Post-Hoc (Bonferroni)	DT-0 vs. DT-1	d=0.829, 95%CI[−0.133,−0.026]	0.002
Post-Hoc (Bonferroni)	DT-0 vs. DT-3	d=0.580, 95%CI[−0.164,−0.003]	0.040
Post-Hoc (Bonferroni)	DT-1 vs. DT-2	d=0.109, 95%CI[−0.039,0.058]	1.000
**CMC V_SHANK**			
LMM	Main Effect: Session	F(1,206)=36.00, β=0.007	<0.001
LMM	Interaction: Session × Task	F(1,206)=3.46, β=−0.004	0.073
RM-ANOVA	Main Effect: Condition	F(3,87)=20.08, $\eta_{p}^{2}=0.445$	<0.001
Post-Hoc (Bonferroni)	DT-0 vs. DT-1	d=1.142, 95%CI[−0.054,−0.018]	<0.001
Post-Hoc (Bonferroni)	DT-0 vs. DT-3	d=1.025, 95%CI[−0.071,−0.021]	<0.001
Post-Hoc (Bonferroni)	DT-1 vs. DT-2	d=−0.107, 95%CI[−0.016,0.011]	1.000

**Table 2:** Summary of the statistical analysis including the Linear Mixed Model (LMM) for the Task-Evoked Pupillary Response (TEPR) learning trend and the Friedman test for condition-based pupil dilation adaptation.

Statistical Test	Effect/Comparison	Test Statistic	*p*-Value
**Pupil Dilation (z-score normalized)**			
Friedman Test	Main Effect: Condition	$\chi^{2}\left(7\right)=81.41,\;W=0.431$,	<0.001
Post-Hoc (Wilcoxon)	ST-1 vs. DT-0	r=0.803	<0.001
Post-Hoc (Wilcoxon)	ST-1 vs. ST-2	r=0.498	0.0215
Post-Hoc (Wilcoxon)	All DT cond. (pairwise)	−0.089<r<0.071	0.681<p<0.927 (ns)
Post-Hoc (Wilcoxon)	ST-1 vs. ST-3	r=−0.360	0.1023 (ns)
**Task-Evoked Pupillary Response (TEPR)**			
LMM	Main Effect: Session	t(118)=2.26, β=−0.0058	0.025

## Data Availability

The data presented in this study are available on request from the corresponding author. The data are not publicly available due to privacy and ethical restrictions regarding human subjects, as mandated by the Ethics Committee for University Research of the University of Brescia. All data are stored anonymized in a dedicated secure database.
